# Survey of the training and use of echocardiography and lung ultrasound in Australasian intensive care units

**DOI:** 10.1186/s13054-016-1444-9

**Published:** 2016-10-24

**Authors:** Yang Yang, Colin Royse, Alistair Royse, Kacey Williams, David Canty

**Affiliations:** 1 Ultrasound Education Group, Department of Surgery, The University of Melbourne, Melbourne, Victoria Australia; 2 Intensive Care Unit, Western Health, Eastern Health, Epworth Private Hospital, Melbourne, Victoria Australia; 3 Department of Anaesthesia, St. Michael’s Hospital, Toronto, Ontario Canada; 4 Department of Anaesthesia, The Royal Melbourne Hospital, Melbourne, Victoria Australia

## Introduction

Transthoracic echocardiography (TTE) and focused cardiac ultrasound (FCU) are now considered essential skills and a requirement of training for physicians working in the intensive care unit (ICU) [1]. TTE is a feasible and safer alternative to transoesophageal echocardiography (TOE), even after cardiac surgery [2]. Acquiring competency in TTE during an already over-full curriculum is a challenge. Furthermore, lung ultrasound (LU) is becoming established for bedside diagnosis of acute respiratory pathology [3]. The current level of practice and training in TTE, TOE and LU in ICU is not yet reported. We surveyed the 114 ICUs accredited for ICU training in Australasia to determine the current prevalence of practice and training in TTE, TOE, and LU and to identify perceived obstacles in practice and training. After ethics approval, a web-based survey of 14 Multiple Choice Questions was submitted to the Directors of ICU accredited for training.

## Findings

Out of 114 ICUs, 69 (61 %) completed the survey, including 23 (33 %) that admitted cardiothoracic surgical patients and 44 (67 %) that did not. The proportion of ICUs performing TTE (94 %) and LU was high (83 %) but the use of TOE was low (33 %). The proportion of ICU consultants performing TTE, LU, and TOE was lower (42 %, 51 %, and 10 %), respectively. The level of expertise (diagnostic versus focused) was highest in TTE (32 %) compared with TOE (22 %) and LU (12 %).

The proportions of intensivists untrained in TTE and LU was 41 % and 30 %, respectively. Perceived barriers included lack of organized training (38 %) and time for training (25 %). Other barriers included a perceived lack of need for training (18 %), insufficient equipment (14 %), and resistance from other ultrasound providers (4 %). The most commonly reported training programs were tertiary courses, such as provided by the Australasian Society of Ultrasound in Medicine (68 %) and University of Melbourne (59 %), rather than board examinations or hands-on workshops.

We conclude that although TTE and LU are used frequently in Australasian teaching ICUs, many ICU physicians are yet to be trained due to lack of ICU training programs and time for training. Although tertiary courses are popular and provide training to diagnostic level, they are lengthy and depend on trainers and patient caseload and are not, therefore, scalable. An attractive alternative is to begin training in medical school and to train more physicians in basic ultrasound with shorter, more efficient, and hands-on courses utilizing the internet and ultrasound simulators [1], advancing to a diagnostic level only if required.

## Additional file

Additional file 1: Qualtrics Survey Software. (PDF 138 kb)

## Abbreviations

FCU: Focused Cardiac Ultrasound
ICU: Intensive Care Unit
LU: Lung Ultrasound
TOE: Transoesophageal Echocardiography
TTE: Transthoracic Echocardiography
