# Supplementary material for: Survey of the training and use of echocardiography and lung ultrasound in Australasian intensive care units
Source: Crit Care. 2016 Oct 24;20:339. doi: 10.1186/s13054-016-1444-9 (PMC5075752; doi:10.1186/s13054-016-1444-9)
Supplement: Additional file 1: — Qualtrics Survey Software. (PDF 138 kb) [file 13054_2016_1444_MOESM1_ESM.pdf]

Default Question Block

.

INFORMATION AND CONSENT

THE ROYAL MELBOURNE HOSPITAL, DEPARTMENT OF SURGERY

1. Participant Information and Consent Form for Quality Assurance Project:
Survey of current ultrasound and echocardiography practice in accredited Australasian intensive care units

Investigators:
Principal Researchers: Dr David Canty, Dr Kacey Williams and Dr Yang Yang,
Associated Researchers: Associate Researchers: Professor Colin Royse, Professor Alistair Royse

You are invited to take part in this quality assurance project. Participation is voluntary and you may decline if you wish. If you decide to take part and later change your mind, you are free to withdraw from the project at any stage.

Please read this information and ask questions if you need more information. Once you understand what the project is about and if you agree to take part in it, please proceed to complete the short questionnaire provided.

2. Purpose
This survey seeks to audit the practices and training in bedside point-of-care echocardiography and lung ultrasound performed by consultant intensvists to facilitate the management of critically ill patients.

You are invited to participate in this research project because you are a the Head or acting Head of Department at an accredited Australasian ICU.

3. Procedures
Participation in this project will involve the completion of a short questionnaire which should only take 1-10 minutesof your time.

4. Possible Risks (if applicable)
Participation in this project will involve no risks and minimal inconvenience

5. Privacy, Confidentiality and Disclosure of Information
Any information obtained in connection with this project and that can identify you will remain confidential. It will only be disclosed with your permission, except as required by law. In any publication of the results of the project, information will be provided in such a way that you cannot be identified.

6. Results of Project
Upon completion of the study we intend to present the results of the survey in the form of a presentation or scientific paper for submission to a peer-reviewed journal. Results of the survey will be made available to participants upon request.

7. Other Issues
If you have any complaints about any aspect of the project, the way it is being conducted or any questions about your rights as a research participant, then you may contact

Name: Ms Angela Gray
Position: Assistant Manager, Office for Research
Telephone: (03) 9342 7550

- ○ I have read the the consent form and **I agree** to participate in this survey
- ○ I don't agree to participate in this survey

.

DEFINITIONS

1. Diagnostic or comprehensive echocardiography

Diagnostic echocardiography (TTE or TOE) is a complete systematic examination that is performed according to the guidelines published by the American Society of Echocardiography.(1, 2)

2. Focused cardiac ultrasound (FCU)

FCU is a focused examination of the cardiovascular system performed by a physician by using ultrasound as an adjunct to the physical examination to recognize specific ultrasonic signs that represent a narrow list of potential diagnoses in specific clinical settings. Focused cardiac ultrasound is an abbreviated, goal-directed form of transthoracic echocardiography (3) or transoesophageal echocardiography (4) such uses included assessment and monitoring of left ventricular volume and function.

3. Diagnostic lung ultrasound

Diagnostic lung ultrasound is a comprehensive examination of the pleural and parenchyma (5).

4. Focused lung ultrasound

Focused lung ultrasound is a focused examination of the respiratory system performed by a physician by using ultrasound as an adjunct to the physical examination to recognize specific ultrasonic signs that represent a narrow list of potential diagnoses in specific clinical settings such as pleural effusion and interstitial syndrome. Focused lung ultrasound is an abbreviated, goal-directed form of diagnostic lung ultrasound.

References

1. Hahn RT, Abraham T, Adams MS, et al. Guidelines for performing a comprehensive transesophageal echocardiographic examination: recommendations from the American Society of Echocardiography and the Society of Cardiovascular Anesthesiologists. Journal of the American Society of Echocardiography : official publication of the American Society of Echocardiography. 2013;26(9):921-64.

2. Lang RM, Bierig M, Devereux RB, et al. Recommendations for chamber quantification. European journal of echocardiography : the journal of the Working Group on Echocardiography of the European Society of Cardiology. 2006;7(2):79-108.

3. Spencer KT, Kimura BJ, Korcarz CE, et al. Focused cardiac ultrasound: recommendations from the American Society of Echocardiography. Journal of the American Society of Echocardiography : official publication of the American Society of Echocardiography. 2013;26(6):567-81.

4. Reeves ST, Finley AC, Skubas NJ, et al. Special article: basic perioperative transesophageal echocardiography examination: a consensus statement of the American Society of Echocardiography and the Society of Cardiovascular Anesthesiologists. Anesthesia and analgesia. 2013;117(3):543-58.

5. Volpicelli G, Elbarbary M, Blaivas M, et al. International evidence-based recommendations for point-of-care lung ultrasound. Intensive care medicine. 2012;38(4):577-91.

Q1. **Regarding performance of transthoracic echocardiography (TTE) in your ICU:**

- **None:** All TTE is performed by another service such as cardiology or radiology. i.e. ICU staff do not perform any TTE.
- **Focused TTE:** Some TTE is performed by ICU consultant staff but is restricted to focused TTE, such as for assessment of ventricular volume and function. Diagnostic TTE is referred to another service such as cardiology or radiology.
- **Diagnostic TTE:** Some diagnostic TTE is performed by ICU consultant staff.

Q2.

How many consultant ICU staff (fractional or full-time) are employed in your ICU?

Q3.

What proportion (%) of your consultant ICU staff perform focused or diagnostic TTE?

Q4.

Regarding performance of transoesophageal echocardiography (TOE) in your ICU:

Definitions

1. Diagnostic or comprehensive echocardiography

Diagnostic echocardiography (TTE or TOE) is a complete systematic examination that is performed according to the guidelines published by the American Society of Echocardiography.(1, 2)

2. Focused cardiac ultrasound (FCU)

FCU is a focused examination of the cardiovascular system performed by a physician by using ultrasound as an adjunct to the physical examination to recognize specific ultrasonic signs that represent a narrow list of potential diagnoses in specific clinical settings. Focused cardiac ultrasound is an abbreviated, goal-directed form of transthoracic echocardiography (3) or transoesophageal echocardiography (4) such uses included assessment and monitoring of left ventricular volume and function.

- **None:** All TOE is performed by another service such as cardiology, radiology or anaesthesia. i.e. ICU staff do not perform any TOE.
- **Miniature TOE** monitoring (eg. hTEE Imacor) is performed in your ICU.
- **Focused TOE:** Some TOE is performed by ICU consultant staff but is restricted to focused TOE, such as for assessment of ventricular volume and function. Diagnostic TOE is referred to another service such as cardiology or radiology (eg. valvular quantification or aortic dissection).
- **Diagnostic TOE:** Some diagnostic TOE is performed by ICU consultant staff.

Q5.

What proportion (%) of your consultant ICU staff perform focused or diagnostic TOE?

Q6.

Regarding performance of lung ultrasound in your ICU:

1. **Diagnostic lung ultrasound**

Diagnostic lung ultrasound is a comprehensive examination of the pleural and parenchyma (5).

2. **Focused lung ultrasound**

Focused lung ultrasound is a focused examination of the respiratory system performed by a physician by using ultrasound as an adjunct to the physical examination to recognize specific ultrasonic signs that represent a narrow list of potential diagnoses in specific clinical settings such as pleural effusion and interstitial syndrome. Focused lung ultrasound is an abbreviated, goal-directed form of diagnostic lung ultrasound.

- **None:** All lung ultrasound is performed by another service such as respiratory medicine or radiology. i.e. ICU staff do not perform any lung ultrasound.
- **Focused lung ultrasound:** Some lung ultrasound is performed by ICU consultant staff but is restricted to focused lung ultrasound, such as for assessment of pleural effusions and interstitial syndrome. Diagnostic lung ultrasound is referred to another service such respiratory medicine or radiology.
- **Diagnostic lung ultrasound:** Some diagnostic lung ultrasound is performed by ICU consultant staff.

Q7.

What proportion (%) of your consultant ICU staff perform focused or diagnostic lung ultrasound?

Q8. **Training of echocardiography for ICU staff**:

- **No current** organised (and regular) echocardiography training program for ICU staff
- **Out-sourced:** Echocardiography training program is provided for ICU staff from another service, eg. cardiology
- **In-house:** Echocardiography training program is provided by ICU staff

Q9. **Training of lung ultrasound for ICU staff**:

- **No current** organised (and regular) lung ultrasound training program for ICU staff
- **Out-sourced:** lung ultrasound training program is provided for ICU staff from another service, eg. respiratory
- **In-house:** Lung ultrasound training program is provided by ICU staff

Q10.

What training/ accreditation pathways are recommended for your ICU staff? (do not include workshops)

- Australian Society of Ultrasound in Medicine - eg. DDU, CCPU, DMU
- University of Melbourne Graduate Courses -eg. certificate, diploma, masters, simulator courses
- National Board of Echocardiography exams (North America) - eg. TOE PTeeXAM, TTE ASCeXAM
- British Society of Echocardiography exams (BSE)
- European Society of Echocardiography (ESE) exams
- Other, please specify:

Q11.

What do believe is the principal impediment to developing echocardiography practice in your ICU? PLEASE RANK IN ORDER OF PRIORITY 1 to 5 BY SELECTING AND DRAGGING THE ITEM UP OR DOWN.

- **Not required:** Currently a full (including after hours) echocardiography service is provided by another service, eg. cardiology or radiology.
- **Equipment**
- **Training**
- **Lack of cooperation** with other providers of echocardiography, eg. cardiology
- **Other**- PLEASE SPECIFY

. Please press Next to complete this study.
